# Non-Coding RNAs and Hepatitis C Virus-Induced Hepatocellular Carcinoma

**DOI:** 10.3390/v10110591

**Published:** 2018-10-30

**Authors:** Marie-Laure Plissonnier, Katharina Herzog, Massimo Levrero, Mirjam B. Zeisel

**Affiliations:** 1 Cancer Research Center of Lyon (CRCL), UMR Inserm 1052 CNRS 5286 Mixte CLB, Université de Lyon 1 (UCBL1), 69003 Lyon, France; marie-laure.plissonnier@inserm.fr (M.-L.P.); massimo.levrero@inserm.fr (M.L.); 2 Inserm, U1110, Institut de Recherche sur les Maladies Virales et Hépatiques, 67000 Strasbourg, France; katharina.herzog@etu.unistra.fr; 3 Université de Strasbourg, 67000 Strasbourg, France; 4 Hospices Civils de Lyon, Service d’Hépato-Gastroentérologie, 69004 Lyon, France

**Keywords:** hepatocellular carcinoma, hepatitis C virus, microRNA, long non-coding RNA

## Abstract

Hepatitis C virus (HCV) infection is a worldwide health problem and is one of the main causes of chronic hepatitis, liver cirrhosis, and hepatocellular carcinoma (HCC). Despite recent improvements, effective treatments for HCC are still missing and new tools for early detection are needed. Non-coding RNAs (ncRNAs) have emerged as important regulators of gene expression and key players in human carcinogenesis, including HCC. Aberrant expression of ncRNAs is associated with HCC metastasis, invasion, dissemination, and recurrence. This review will focus on the recent advances in ncRNA expression profiles, their dysregulation in HCV-related HCC, and the clinical perspective of ncRNA signatures for the early detection of HCC.

## 1. Introduction

Despite an overall drop in the incidence of hepatitis C virus (HCV) infection within the past years due to direct-acting antivirals (DAAs), which enable chronic hepatitis C (CHC) to be cured, an estimated 71 million individuals are still chronically infected by HCV worldwide [1]. HCV infection is a major risk factor of HCC and associated with 20% of cases of liver cirrhosis, which is functional decompensation leading to hepatocellular carcinoma (HCC) [2]. Other major HCC etiologies include chronic hepatitis B virus (HBV) infection, alcohol abuse, and metabolic causes. In HCV-infected patients, HCC development is a consequence of fibrosis progression and occurs after the establishment of cirrhosis [3]. According to the GLOBOCAN series of the International Agency for Research on Cancer in 2012 [4], HCC is the fifth most common cancer and the second cause of cancer death worldwide.

HCC is a poor prognosis disease. Although the risk of developing HCC can be reduced in patients by treatment of the underlying cause, e.g., by viral clearance or abstinence from alcohol, strategies to prevent cancer development in patients with advanced fibrosis and/or established cirrhosis are still lacking. Early diagnosis increases the chance of effective therapy, but the detection of small liver tumours by ultrasound is challenging and currently there is no reliable serum biomarker that can be used in surveillance programmes. Patients with advanced HCC carry a very poor prognosis, with an expected survival of four to six months and despite recent improvements, treatment options for HCC remain scarce. According to the clinical practice guidelines from the European Association for the Study of the liver (EASL), patients eligible for curative treatment can undergo surgical resection, radiofrequency ablation, or liver transplantation [5]. Tumour recurrence remains the major cause of death in HCC patients following loco-regional ablation and liver resection. Intermediate (stage B according to the Barcelona-Clínic Liver Cancer (BCLC) classification [5]) HCCs have been shown to benefit from trans-arterial chemoembolization (TACE) [5]. The first-line treatments for patients with advanced HCC (BCLC stage C) [5], which are not eligible for curative treatment, are the multikinase inhibitors sorafenib and lenvatinib, which increase survival by approximately three months [6]. Phase III clinical trials with regorafenib or cabozantinib as second-line treatment for HCC patients undergoing tumour progression after sorafenib (RESORCE, CELESTIAL) showed an extension of overall survival by 10 months [7,8]. Novel treatment options and prognostic tools are required to improve the management of patients with HCC.

Among the risk factors of HCC, HCV is a positive-strand RNA virus [9,10] that does not integrate into the host genome. It encodes a large polyprotein of about 3000 amino acids from a single open reading frame which is processed into three structural (core, E1, and E2) and seven non-structural (p7, NS2, NS3, NS4A, NS4B, NS5A, and NS5B) proteins [11]. HCV proteins interact with many host-cell factors well beyond their roles in the viral life cycle and are notably involved in cell signalling, transcription, cell proliferation, apoptosis, vesicular trafficking, and translational regulation [12]. HCV proteins contribute to HCC by modulating pathways that promote the malignant transformation of hepatocytes through the accumulation of genetic damages and epigenetic dysregulation [13,14,15]. For instance, the HCV core protein sensitizes host cells to TRAIL-induced cell apoptosis by activating the CK1α-p53-Bid dependent pathway in human hepatoma cells [16] or is able to suppress the p53-dependent apoptosis induced by the all-*trans* retinoic acid (ATRA), the most biologically active metabolite of vitamin A [17]. Moreover, the HCV core protein binds to several tumour suppressor proteins, including p53, p73, and pRb [18,19]. Despite the development of DAAs enabling an HCV cure and reducing the risk of liver complications [20,21], a long-term risk of HCC remains in cirrhotic patients, even after achieving a sustained virological response (SVR) [22]. Of note, while some reports published within the past two years have raised concerns about a potential higher risk of post-SVR HCC in patients treated with DAAs [23,24,25] compared to patients treated with the previous standard-of-care interferon and ribavirin, several subsequent reports—including large multicenter studies—have failed to confirm this [21,26,27,28,29,30,31,32,33]. 

Liver carcinogenesis is a multistep process driven by chronic inflammation, DNA damage, senescence and telomerase reactivation, chromosomal instability, and epigenetic modifications. All etiologic factors seem to act through similar mechanisms (i.e., point mutations, chromosomal aberrations, epigenetic changes) that converge to affect common pathways. Notably, mutations and chromosomal aberrations have been predominantly found in malignant tumour tissues, whereas the dysregulation of signalling pathways and epigenetic changes are also detected earlier in the natural history of HCC development, at the stage of cirrhosis [34]. Epigenetic changes that include DNA methylation, post-translational histone modifications, and ncRNA-mediated silencing pathways occur early in the development of HCC. Several studies have also identified mutations in a group of chromatin regulators (*ARID1A*, *ARID1B*, *ARID2*, *MLL*, and *MLL3*) in approximately 20% of all tumours, including virus- and alcohol-related HCCs (reviewed in [34]). Modulation of the methylation status of DNA CpG islands and lysines in the histone tails in gene promoters represents important epigenetic alterations in human cancer, including HCC. Next-generation sequencing technology has revealed that many ncRNAs play important roles in biological processes, such as differentiation, proliferation, and cell death [35,36,37], as well as in cancer [38,39]. Based on their length, ncRNAs are classified into small ncRNAs (sncRNAs, less than 200 nucleotides) and long ncRNAs (lncRNAs, more than 200 nucleotides), according to the RNA purification protocol that excludes small RNAs described by Kapranov and colleagues [40]. Aberrant expression of ncRNAs is associated with HCC metastasis, invasion, dissemination, and recurrence [41,42,43,44,45], suggesting that ncRNAs are key players of human carcinogenesis, including HCC. This review will focus on sncRNAs and lncRNAs in HCV-induced HCC.

## 2. Expression and Functions of ncRNAs in HCV-Related HCC

### 2.1. LncRNAs in HCV-Related HCC

LncRNAs are a heterogeneous group of non-coding transcripts more than 200 nucleotides long that are transcribed by RNA polymerase II, 5′ capped, spliced, and polyadenylated [46,47]. The FANTOM project [48] and the GENCODE consortium in the framework of the ENCODE project [49] led to the identification of over 20,000 lncRNAs. In 2015, an analysis of 7256 RNA-seq libraries from tumours, normal tissues, and cell lines reported the identification of 58,648 lncRNA genes [46]. 

LncRNAs are involved in many biological processes, such as proliferation, differentiation and development [47,50]. The dysregulation of lncRNAs significantly contributes to numerous human diseases, especially cancers [51,52], including HCC [47,53]. The structural complexity of lncRNAs offers multiple possibilities for interactions with DNA, RNA, and/or proteins that depend on secondary and tertiary structures. Several lncRNAs are localized to specific cellular compartments, nuclear or cytosolic, in accordance with their biological function. Nuclear lncRNAs can act as guides for chromatin-modifying-complexes or transcription factors. The majority of lncRNAs are in the cytoplasm and often function as regulators of protein levels, either by directly controlling mRNA stability or by acting as competing endogenous RNA [54]. Depending on their genomic location and context, lncRNAs are classified into five categories [50,55]: (1) intergenic lnRNAs, the so-called lincRNAs are transcribed in the intergenic region between two protein-coding genes; (2) sense/intronic lncRNAs, which arise from intronic regions within protein-coding genes; (3) antisense lncRNAs (NATs), when overlapping one or more exons of another transcript on the opposite strand; (4) bidirectional lncRNAs, such as promoter upstream transcripts (PROMPTSs), which are transcribed in the promoter regions of protein-coding genes in a bidirectional manner; and (5) enhancer lncRNAs which are transcribed in the enhancer regions of the genome. Two other ncRNAs can be considered as lncRNAs: circular RNAs (circRNAs), which are created from protein-coding mRNAs or linear ncRNAs that join an upstream 3′ splice site and downstream 5′ splice site to form a covalently closed continuous loop [56]; and pseudogenes, which originate from gene duplication and have acquired various mutations, inducing a loss of their protein-coding capacity [57,58]. Most of these pseudogenes serve as competitive endogenous RNAs (ceRNA) to sequester miRNAs. The *PTEN* pseudogene, *PTENP1*, was the first pseudogene shown to regulate the expression of its parental gene by binding and sequestering *PTEN*-targeting miR-17, miR-19, miR-20a, and miR-21 in prostate cancer [57].

LncRNAs regulate gene expression through different mechanisms, such as epigenetic silencing, splicing regulation, lncRNA-miRNA interaction by sequestering miRNAs, lncRNA-protein interaction, and genetic variation [53,59,60]. The molecular functions of lncRNAs are not well-characterized, but four categories of mode of action have been proposed [39,53]. LncRNAs can function as decoys by binding and titrating away proteins or RNA targets such as miRNAs. This family of lncRNAs can negatively regulate the expression of their target or bind to the transcription binding sites and avoid the fixation of the transcription factor. LncRNAs can also have a guide function: these lncRNAs bind proteins and direct their localization to a specific target. They act, for example, as epigenetic repressors, i.e., HOX transcript antisense intergenic RNA (HOTAIR), or epigenetic activators, such as HOTTIP or H19. Moreover, signal lncRNAs are expressed in a cell-type specific and stage-specific manner and can regulate transcriptional activity or biological pathways by interacting with transcription factors or chromatin-modifying enzymes. Finally, lncRNAs can have a scaffold function. This class of lncRNAs serves as a central platform to bind different molecular components and facilitates their intermolecular interactions.

Many studies have described the role of lncRNAs in HCC (for review see [47,53,61,62,63]). HCC-related lncRNAs participate in diverse biological processes involved in HCC progression, such as cell proliferation, apoptosis, invasion, metastasis, and angiogenesis. In the last decade, hundreds of dysregulated lncRNAs have been characterized in HCC tissues compared with normal tissues [64]. For example, the lncRNA HOTAIR is highly expressed in HCC and is associated with poor prognosis [65] and an increased risk of recurrence and metastasis [66,67]. Recently, lncRNA HULC polymorphisms have been associated with HCC risk and prognosis [68]. Furthermore, Guo and colleagues demonstrated that the lncRNA PVT1 is upregulated in HCC, promoting HCC cell propagation and inhibiting apoptotic cells by recruiting EZH2 [69].

LncRNAs can be differentially expressed depending on the HCC etiology. In 2015, Zhang et al. explored lncRNA expression profiles of 73 tissue samples at different stages of HCV-induced HCC: cirrhotic tissue, dysplastic nodules, and HCC samples compared to healthy liver tissue [70]. The expression of seven lncRNAs (LINC01419, BC014579, AK021443, RP11-401P9.4, RP11-304 L19.5, AF070632, CTB-167B5.2) in preneoplastic lesions and HCC was significantly different. Among these lncRNAs, the lncRNA LINC01419 transcripts were expressed at higher levels in early stage HCC compared to dysplasia and early stage HCC, and were overexpressed in HCV-related HCC when compared with matched non-tumour liver tissues. LncRNA AK021443 levels increase in advanced stage HCC, while lncRNA AF070632 levels decrease in advanced stage HCC. Moreover, computational analysis suggested that LINC01419 and AK021443 regulate cell cycle genes, whereas AF070632 is associated with cofactor binding, oxidation-reduction, and the carboxylic acid catabolic process. LINC01419 and AK021443 could thus promote HCV-related HCC development by modulating the cell cycle progression. In another study conducted by Zhang et al. in 2016 [71], lncRNA hypoxia-inducing factor a (aHIF), Prader Willi/Angelman region RNA 5 (PAR5), and human downregulated expression by HBx (hDREH) were associated with HCV-related HCC since their expressions were significantly downregulated (aHIF and PAR5) or upregulated (hDREH) in tumour vs. non-tumour tissues, but these observations have to be confirmed in a larger patient cohort. Two additional lncRNAs, urothelial carcinoma associated-1 (UCA1) and WD repeat containing antisense to TP53 (WRAP53), have been found to be upregulated in HCC patients with chronic HCV infection [72]. UCA1 upregulation has been shown to increase epithelial-to-mesenchymal transition in HCC via sponging miR-203, thereby activating the expression of transcription factor Snail2 [73] (Table 1). 

### 2.2. SncRNA in HCV-Related HCC

High-throughput sequencing technologies have enabled researchers to uncover different types of sncRNA: small nucleolar RNAs (snoRNAs), piwi-interacting RNAs (piRNAs), and miRNAs. Within the last decade, the role of sncRNAs—and particularly the one of miRNAs—in physiological and pathological processes, including HCC, has been extensively studied [38]. 

#### 2.2.1. Small Nucleolar RNAs (snoRNAs)

SnoRNAs are a class of intermediate-sized ncRNAs of 60 to 300 nucleotides discovered in the nucleolus and able to regulate ribosome maturation and function [75], as well as alternative splicing and gene silencing [76,77]. SnoRNAs are divided into two major highly conserved families based on their structure and main function: box C/D snoRNAs and box H/ACA snoRNAs. Box C/D snoRNAs are responsible for 2′-*O*-methylation of ribosomal RNAs [78], while the second family of snoRNAs guides the pseudouridylation of nucleotides [79]. A third less represented class includes the small Cajal body-specific RNAs (scaRNAs) that are associated with Cajal bodies, which are small membrane-less sub-compartments of the nucleus [80]. SnoRNAs are located in introns. They are components of small nucleolar ribonucleoprotein (snoRNPs) complexes along with specific proteins and function as a guide for the post-transcriptional modification of ribosomal RNAs by facilitating rRNA folding and stability [81,82]. The sequences of snoRNAs are responsible for targeting the assembled snoRNPs to a specific target. 

Alterations in snoRNA expression in human cells can affect numerous biological processes, leading to tumorigenesis. Six snoRNAs are well-described to be dysregulated in HCC [83], regardless of the etiological factor. For example, SNORD113-1 is significantly downregulated in HCC-tumour tissues compared with non-tumour tissues; furthermore, a statistically significant association between low-level expression of SNORD113-1 and relapse-free survival was observed, which suggests that downregulation of SNORD113-1 is associated with HCC aggressiveness [84]. Furthermore, a study performed by Fang et al. [85] demonstrated that SNORD126—located within the intron of the *cyclin B1-interacting protein 1 (CCNB1IP1)* gene—was upregulated to a high level in HCC compared with non-tumour tissues. Overexpression of SNORD126 was associated with a shorter survival rate in HCC patients and promoted HCC growth through upregulation of the PI3K-AKT pathway. 

#### 2.2.2. Piwi-Interacting RNAs (piRNAs)

PiRNAs are ncRNAs of 24–30 nucleotides in length that bind to the piwi subfamily of argonaute proteins to form a piRNA-induced silencing complex (piRISC), which inhibits transposon mobilization by both epigenetic and post-transcriptional silencing [86,87]. They are transcribed from regions in the genome that contain transcribed transposable elements and other repetitive elements. Three major PIWI-class proteins (PIWIL1, PIWIL2, and PIWIL4) are involved in a so-called ‘ping-pong’ amplification cycle, creating antisense piRNAs that are capable of repressing the transcript of origin [87]. 

PiRNAs are abundant in the human liver, but no data is available on specific piRNA expression profiles in HCV-related HCC. However, it has been shown by small RNA-seq that an expression pattern of 125 piRNAs clearly differentiates cirrhotic liver from HCC tissues. Interestingly, 24 piRNAs dysregulated in advanced HCC also showed distinctive expression patterns in earlier hepatic lesions, suggesting that these ncRNAs may participate in the carcinogenic process in the liver and could represent new markers of early hepatocarcinogenic lesions [87,88]. The accumulation of piR-LLi-30552 and has-piR-020498 is associated with progression from the dysplasia stage to HCC [88]. Furthermore, Law et al. showed that piR-Hep1 is upregulated in 46.6% of HCC tumours compared to the corresponding adjacent non-tumour liver. piR-Hep1 could play a functional role in hepatocarcinogenesis as it has been shown to promote cell viability, motility, and invasiveness, with a concomitant increase in the level of active AKT phosphorylation [89].

#### 2.2.3. MicroRNAs (miRNAs)

MiRNAs are an important class of ncRNAs of 18–25 nucleotides that regulate gene expression at the post-transcriptional level. MiRNAs bind to the 3′ untranslated region (3′UTR) of complementary sequences of mRNAs to mediate mRNA deadenylation or translation blockage [90]. MiRNAs are estimated to regulate the translation of more than 60% of protein-coding genes: a single miRNA can target hundreds of mRNAs, thereby affecting a broad network of genes [91]. 

Biogenesis of miRNAs takes place through a multistep process [92]. miRNAs are most commonly transcribed in the nucleus by the RNA polymerase II (Pol II). Monocistronic or polycistronic primary miRNA transcripts (pri-miRNAs) are processed into precursor miRNAs (pre-miRNAs) by the DGCR8-Drosha complex and exported to the cytoplasm by exportin 5. These pre-miRNAs undergo cleavage by the endoribonuclease called Dicer that produces a miRNA duplex. These molecules are loaded by the Dicer–TARBP2 (TAR RNA-binding protein 2; also known as TRBP) complex into a member of the argonaute protein subfamily to form the RNA-induced silencing complex (RISC), of which argonaute proteins are the catalytic endonuclease components. RISC directs the regulation of mRNAs by recognizing a complementary sequence in the targeted mRNAs. Translation of mRNAs into proteins is repressed by miRNAs by two main means: mRNA degradation and the inhibition of translation initiation [93].

Several studies have investigated the role of miRNAs in various biological processes [38,39,94,95,96], including proliferation, differentiation, angiogenesis, apoptosis, and development. Abnormal expression levels of miRNAs have been described in inflammation, Alzheimer’s disease, cardiovascular disease, cancer, and viral infection, including HCV infection [38,39,42,54,97]. Of note, several studies revealed an association between the dysregulation of miRNAs and HCC carcinogenesis, including HCV-related HCC [42,98,99]. For example, miR-221 that modulates different pathways involved in the proliferation of tumour cells, survival, and metastasis [100,101] is frequently upregulated in HCC with advanced tumour stages and associated with poor prognosis, irrespective of the HCC etiology. In contrast, other miRNAs have been shown to be specifically deregulated in HCV-induced HCC. Using a microarray analysis of liver tissue samples, Diaz et al. identified 18 miRNAs specifically expressed in HCV-related HCC, including 15 miRNAs that had already been reported in previous studies and three miRNAs (miR-497, miR-1269, and miR-424-3p) which had not been previously described to be modulated in HCV-related HCC (Table 2) [42]. 

Furthermore, it has been shown that the expression of oncogenic miR-155 is increased in patients infected with HCV and this promotes hepatocyte proliferation and tumorigenesis by activating the Wnt signalling pathway [102]. Likewise, miR-135a-5p was shown to be upregulated in HCV-infected patients and to promote the HCV-induced STAT3 transcriptional program in the liver of patients by suppressing its regulator protein tyrosine phosphatase receptor delta (PTPRD), resulting in the malignant progression of liver disease [103]. These studies underscore the functional role that miRNAs may play in the pathogenesis of HCV-induced HCC.

**Figure 1:**
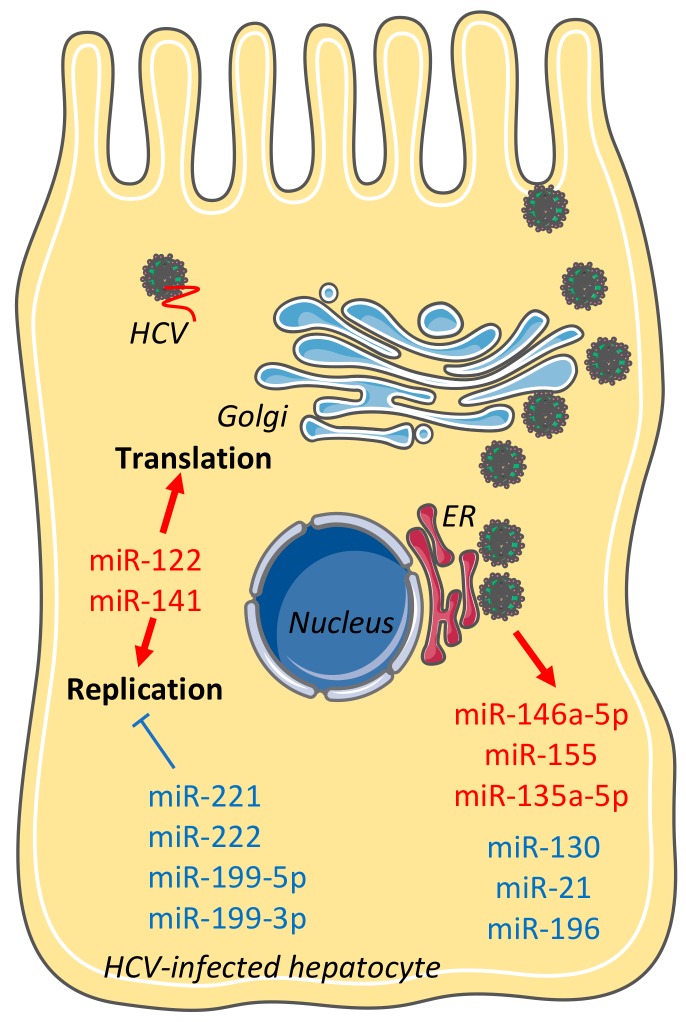
Impact of cellular miRNAs on the HCV life cycle and their contribution to HCV-induced HCC. Impact of individual miRNAs whose expression is modulated upon HCV infection on steps of the HCV life cycle (entry, translation, replication, assembly, and LVP release) are shown. miRNAs in blue display a proviral effect; miRNAs in red have an antiviral effect. HCV: hepatitis C virus. LVP: lipoviral particle. ER: endoplasmic reticulum. Golgi: Golgi apparatus. Images were adapted from SMART (Servier Medical Art).

The study of the role of miRNAs in HCV-induced HCC is particularly interesting since there is a tight interplay between HCV, miRNAs, and hepatocyte metabolic pathways that contributes to liver disease development. One of the hallmarks of HCV replication is its dependency on miR-122, the most abundant miRNA in the liver. miR-122 plays a major role in liver physiology by regulating metabolic pathways and as a tumour suppressor (for review see [104]). It has been shown that HCV sequesters miR-122 from its endogenous mRNA targets, thereby leading to their derepression and liver carcinogenesis [105]. Several other miRNAs that have a dual role in both the HCV replication cycle and in liver disease development have been reported, underscoring the tight interplay between HCV and miRNAs in the liver (Figure 1). 

A recent study that comprehensively analysed miRNAs modulated upon HCV infection showed that several of these miRNAs were known to regulate inflammation, fibrosis, and cancer development [106]. Among the miRNAs whose expression increased during HCV infection was miR-146a-5p, which has been associated with a modulation of the proteasome pathway, fatty acid, and anaerobic energetic metabolism. These regulatory pathways are both in favour of HCV infection (Figure 1) and liver disease development by promoting liver inflammation and HCC [106]. Furthermore, it has been shown that HCV infection modulates miR-196, miR-130, and miR-21 that are able to regulate type I IFN signalling pathways to overcome their antiviral activity [45] (Figure 1). These data are in line with miRNA expression profiles (differential expression of 19 miRNAs, including miR-124b, miR-34c, and miR-23a) associated with HCV infection [41]. Analysis of targeted genes using infection-associated miRNAs revealed that the pathways related to the immune response, antigen presentation, the cell cycle, proteasome, and lipid metabolism, are activated in the HCV-infected liver, suggesting their implication in HCV-induced liver disease pathogenesis. Interestingly, some of these miRNAs also contribute to the modulation of HCV infection. For example, miR-141 that is upregulated in HCV-infected cells has been shown to downregulate the DLC-1 (Rho GTPase) tumour suppressor and to be required for HCV replication [44] (Figure 1). Finally, miR-199-5p/miR-199-3p, miR-221, and miR-222, whose expression is correlated with fibrosis in HCV-infected patients, have been shown to reduce HCV RNA replication by binding the stem loop II region of HCV 5′ UTR [107]. Of note, while DAA therapy has been reported to modulate hepatic miRNA expression, no significant changes in the expression of miRNAs that have been previously associated with a pro- or antiviral effect on HCV were shown, except an increase in miR-122 expression [108].

## 3. Conclusions and Perspectives

### 3.1. ncRNAs as Novel Biomarkers for Detection of HCV-Induced HCC

By contributing to the regulation of the HCV life cycle, liver disease development, and carcinogenesis, ncRNAs play a major role in CHC. Most CHC patients are asymptomatic for many years, and HCC usually develops after several decades of HCV infection [5]. The long latency period between initial HCV infection and HCC development provides an important time window of opportunity for individuals to be monitored for disease progression and intervention. Since early diagnosis increases the chance of effective therapy, patients with cirrhosis are enrolled in periodic ultrasound-based surveillance programmes [109]. However, given the challenge of detecting small liver tumours using ultrasound and the absence of a robust HCC serum marker, reliable non-invasive biomarker(s) would be most helpful for determining HCC risk and/or detecting HCC at early stages. 

**Figure 2:**
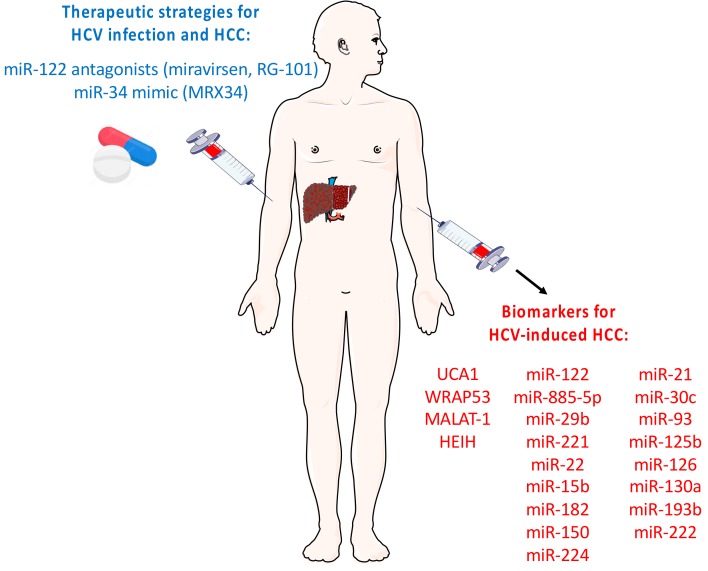
Schematic representation of the potential use of ncRNAs as therapeutic targets for HCV infection and HCC or biomarkers for HCV-induced HCC. MiR-122 antagonists miravirsen and RG-101 have been shown to lead to a dose-dependent reduction of viral RNA in CHC patients [108,109]. miR-34 mimic (MRX34, Mirna Therapeutics) has been administred to patients with primary liver cancer, but this trial was abrogated due to immune-related adverse effects. LncRNAs and miRNAs that have been suggested as biomarkers for HCV-induced HCC are also indicated [42,72,74,111]. HCV: hepatitis C virus. HCC: hepatocellular carcinoma. Images were adapted from SMART (Servier Medical Art).

Molecular signatures using ncRNA expression profiles have been described as potential predictive or prognostic biomarkers of HCC and circulating ncRNAs hold promise as biomarkers for the (early) detection of HCC. In association with alpha-fetoprotein (AFP, the currently most widely used diagnostic HCC serum marker), UCA1 and WRAP53 have been suggested as diagnostic and prognostic markers of HCV-induced HCC [72] (Figure 2). Indeed, a high expression of serum UCA1 was significantly associated with a high tumour grade, large tumour size, positive vascular invasion, and advanced TNM stage in HCC patients [110]. Furthermore, two other lncRNAs might hold promise as potential biomarkers for HCV-related HCC: MALAT1 and HEIH (Figure 2). Indeed, it has been reported that MALAT1 in combination with AFP serum levels might indicate a worse liver failure score in HCV-related HCC patients [111] and lncRNA HEIH expression in serum and exosomes appeared to be increased in HCV-related HCC patients in contrast to patients with CHC or HCV-induced cirrhosis [74]. Further studies using different patient cohorts are needed to assess the potential of lncRNAs as HCC biomarkers. Several panels of miRNAs have also been suggested as potential HCC biomarkers in HCV-infected patients. For example, Zekri et al. identified an miRNA panel composed of miR-122, miR-885-5p, and miR-29b in association with AFP as a new biomarker for the early detection of HCC in a normal population, while another miRNA panel composed of miR-122, miR-885-5p, miR-221, and miR-22 in association with AFP provided a high diagnostic accuracy for the early detection of HCC in cirrhotic patients [112]. Furthermore, the potential of circulating miR-15b and miR-122, as well as miR-182 and miR-150, have been suggested as biomarkers to assess HCC risk in cirrhotic HCV-infected patients [113] and for the detection of cirrhosis progression and HCC in a cohort of HCV-infected Egyptian patients [114], respectively. Moreover, Okajima et al. [115] showed that circulating miR-224 could be a novel biomarker for the detection of primary and recurrent HCC. Finally, a panel of nine liver-associated miRNAs (miR-21, miR-30c, miR-93, miR-122, miR-125b, miR-126, miR-130a, miR-193b, and miR-222) could discriminate healthy individuals from patients with HCV-related HCC [116] (Figure 2). Further patient cohort studies are required to assess the utility of these miRNAs for CHC patient surveillance programmes. 

### 3.2. ncRNAs as Novel Therapeutic Targets of HCV-Induced HCC

In addition to their potential role as biomarkers, ncRNAs may also represent therapeutic targets (Figure 2). Several studies demonstrated in vivo proof-of-concept of using ncRNA/ncRNA antagonists to modulate gene expression, including in the liver [117,118,119]. LncRNAs that act as transcriptional enhancers might be used to increase gene expression, as demonstrated by the use of “antagoNATs” (oligonucleotides or siRNA designed to inhibit NATs) [119]. Another therapeutic option is to target miRNAs by using miRNA antagonists/antisense oligonucleotides to sequester miRNAs. The feasibility of such strategies for targeting the human liver is demonstrated by clinical trials in CHC patients having shown a dose-dependent prolonged reduction of viral RNA in patients treated with miR-122 antagonists miravirsen or RG-101 [117,118] (Figure 2). Furthermore, another clinical trial tested a nanoliposome-incorporated miR-34 mimic (MRX34, Mirna Therapeutics) in patients with primary liver cancer (Figure 2). However, this trial was abrogated due to immune-related adverse effects. Further investigations are required to fully characterize the biological functions of ncRNAs and to understand the impact of ncRNA modulation in liver carcinogenesis in order to develop new clinical therapies.

## Figures and Tables

**Table 1:** Role of lncRNAs in HCV-induced HCC. The expression of lncRNAs that have been associated with HCV-induced HCC, as well as their biological function, are shown. lncRNAs that have been reported to be uniquely modulated in HCC induced by HCV, but not in HCC induced by another etiological factor, are highlighted in bold.

lncRNA	Expression in HCV-Induced HCC	Molecular Mechanism for HCV-Induced HCC	References
HOTAIR	↑	Epigenetic repression	[66,67]
HULC	↑	Polymorphism	[68]
PVT1	↑	Cell cycle progression	[69]
**LINC01419**	↑	**Regulation of cell cycle genes**	**[70]**
**BC014579**	↑	**unknown**	**[70]**
**AK021443**	↑	**Regulation of cell cycle genes**	**[70]**
**RP11-401P9.4**	↑	**unknown**	**[70]**
**RP11-304 L19.5**	↑	**unknown**	**[70]**
**AF070632**	↓	**Cofactor binding and catabolic processes**	**[70]**
**CTB-167B5.2**	↓	**unknown**	**[70]**
**aHIF**	↓	**unknown**	**[71]**
**PAR5**	↓	**unknown**	**[71]**
LINC01152	↓	unknown	[71]
TMEVPG1	↓	unknown	[71]
BC017743	↑	unknown	[71]
BC043430	↑	unknown	[71]
PCNA-AS1	↑	unknown	[71]
UFC1	↑	unknown	[71]
ZEB1-AS1	↑	unknown	[71]
hDREH	↑	unknown	[71]
UCA1	↑	Control of gene expression (target: miR-203)	[72]
WRAP53	↑	unknown	[72]
MALAT1	↑	Regulation of splicing processes	[73]
HEIH	↑	Cell proliferation	[74]

**Table 2:** miRNA-specific signature of HCV-induced HCC. The expression of miRNAs that have been associated with HCV-induced HCC [42], as well as their biological function, are shown.

miRNA	Expression in HCV-Induced HCC	Molecular Mechanism for HCV-Induced HCC
mir-1269	↑	Increase of proliferation
mir-224	↑	Increase of proliferation
mir-452	↑	Increase of proliferation, migration and invasion
mir-224-3p	↑	unknown
mir-224-5p	↑	unknown
mir-221	↑	Increase of proliferation and invasion
mir-497	↓	Inhibition of proliferation, induction of apoptosis
mir-214	↓	Inhibition of proliferation, migration and invasion
mir-195	↓	Inhibition of proliferation and EMT
mir-130a	↓	Inhibition of proliferation, migration and invasion
mir-125a-5p	↓	Inhibition of proliferation
mir-125b-5p	↓	Inhibition of proliferation
mir-424-3p	↓	unknown
mir-139-3p	↓	Inhibition of proliferation and metastasis
mir-139-5p	↓	Inhibition of EMT, migration and invasion
mir-199b-3p	↓	unknown
mir-199a-3p	↓	Inhibition of proliferation, migration, invasion and angiogenesis
mir-199a-5p	↓	Inhibition of proliferation, migration and invasion
